# Identifying Predictors of Weight‐Related Outcomes With Fixed‐Dose, Extended‐Release Naltrexone and Bupropion Among Treatment‐Adherent Patients in Phase 3 COR Trials: A Treatment Target Analysis

**DOI:** 10.1002/osp4.70159

**Published:** 2026-06-16

**Authors:** Donna H. Ryan, Angela Fitch, Robert F. Kushner, Jena S. Tronieri, Christopher D. Still, Caroline M. Apovian

**Affiliations:** ^1^ Pennington Biomedical Research Center Baton Rouge Louisiana USA; ^2^ knownwell Needham Massachusetts USA; ^3^ Northwestern University Feinberg School of Medicine Chicago Illinois USA; ^4^ University of Pennsylvania Perelman School of Medicine Philadelphia Pennsylvania USA; ^5^ Geisinger Commonwealth School of Medicine Danville Pennsylvania USA; ^6^ Brigham and Women's Hospital Center for Weight Management and Wellness Boston Massachusetts USA

**Keywords:** fixed‐dose extended‐release combination of naltrexone and bupropion, medication adherence, obesity, pharmacotherapy, predictors, treatment targets

## Abstract

**Objective:**

As patient response to obesity medication can vary, it is crucial to identify both those likely and unlikely to respond to treatment. This post hoc analysis aimed to examine predictive characteristics in patients who achieved weight—related targets with fixed‐dose, extended‐release combination of naltrexone and bupropion (NB‐ER).

**Methods:**

This pooled analysis included patients from three Phase 3, placebo‐controlled COR trials (NCT00532779, NCT00567255, NCT00456521). The impact of baseline characteristics in predicting changes in weight‐related anthropometric targets at 56 weeks in those who completed treatment and were ≥ 90% adherent to NB‐ER was evaluated with univariable and multivariable analyses.

**Results:**

Of the 1692 patients analyzed, similar proportions of patients receiving NB‐ER versus placebo were female and White. Among patients treated with NB‐ER with ≥ 90% adherence, 73.2% achieved ≥ 5% body weight (BW) reduction, 47.6% achieved ≥ 10% BW reduction, 27.8% achieved ≥ 15% BW reduction, 33.7% achieved target body mass index, and 23.7% achieved target waist circumference thresholds. Among the baseline characteristics assessed, female sex and White race most consistently predicted the likelihood of achieving the target outcomes.

**Conclusion:**

Adherence to NB‐ER was associated with achieving anthropometric targets; however, the ability to predict a successful response in advance of NB‐ER treatment is still limited.

## Graphical abstract

In this post hoc predictor analysis of the COR trials in patients with overweight or obesity who received fixed‐dose, extended‐release combination of naltrexone and bupropion, cumulative adherence to medication as well as baseline demographics and clinical characteristics were associated with better body weight reduction and anthropometric outcomes generally.

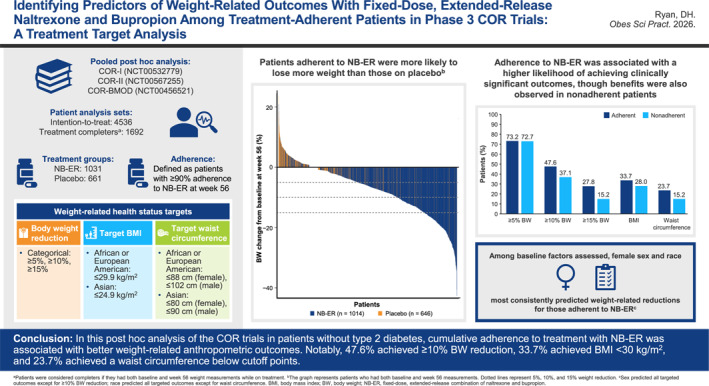

## 1 Introduction

There is significant variability in individuals' responses to weight reduction treatments, including with obesity medications (OMs) [1, 2, 3, 4]. Therefore, identifying characteristics of both those likely and those unlikely to respond to treatment is important to ensure that patients who will benefit most receive treatment and to minimize exposure and economize resources in those who will not.

When identifying potential predictors of treatment response, treat‐to‐target outcomes including anthropometric indicators beyond body weight (BW) reduction should be considered, as these are surrogates for health improvement. A BW reduction beginning at 5% from baseline is viewed as clinically meaningful, and 5%–10% BW reductions can reduce obesity‐related complications [5, 6, 7]. However, even more BW reduction is needed to improve some comorbidities of obesity. For instance, ≥ 10% BW reduction is suggested to improve symptoms of obstructive sleep apnea [6]. There is growing interest in developing tailored treatment targets of obesity to gauge treatment success consistent with the management of diabetes, hypertension, and dyslipidemia [8]. For example, race‐ and sex‐adjusted waist and race‐adjusted body mass index (BMI) thresholds have been validated to identify risk for diabetes and cardiometabolic diseases [9].

While BMI is widely accepted as a parameter to screen patients for excess adiposity and correlates with overall fat mass, individuals can be miscategorized as having overweight or obesity because BMI only measures body size, not body fat or its distribution [8, 9, 10]. Due to these limitations, current guidelines recommend assessing additional anthropometric measures such as waist circumference, as a waist circumference > 88 cm for female patients and > 102 cm for male patients is associated with increased risk for obesity associated comorbidities, including cancer [8, 10, 11]. This metric better demonstrates the distribution of body fat, which has important health implications [10]. Using multiple anthropometric indicators of adiposity adjusted for the race and sex of each patient in clinical practice presents a more holistic assessment of treatment efficacy on weight‐related health status, including obesity‐related cardiometabolic risk [10].

To date, most evaluations of BW reduction variability in response to OMs focus on percentage BW reduction from baseline rather than efficacy as judged by reaching treatment targets; therefore, a target‐based analysis of easily measurable metrics such as percentage body weight reduction, BMI, and waist circumference collectively could be informative [12, 13, 14]. Due to the variability of BW reduction response seen across different therapeutic approaches, how individual baseline characteristics, including sex and race, contribute to the diverse response to OMs in those most likely to achieve benefit could be crucial information to provide to patients and health care providers [10].

The fixed‐dose, extended‐release combination of naltrexone and bupropion (NB‐ER) is approved in many countries, including by the US Food and Drug Administration and European Medicines Agency, as an adjunct to a reduced calorie diet and increased physical activity to reduce excess BW and maintain reduction long‐term in adults with obesity or overweight [15, 16]. In an earlier evaluation of patients who completed an individual Phase 3 study (COR clinical program), patients receiving NB‐ER versus placebo reported mean 8.8% versus 3.1% BW reductions. However, individual responses to NB‐ER varied, and the potential relationship between adherence to medication and weight reduction was not evaluated [17]. That integrated analysis is notable for identifying a process predictor of response, in that BW reduction at week 16 predicted BW reduction at week 56 with 80% accuracy [17]. More recently, several subpopulations have shown favorable responses to NB‐ER, including patients with obesity who smoke, have depression, or exhibit hedonic eating behaviors [18, 19, 20, 21]. However, further research is required to identify the baseline predictors of successful response to treatments like NB‐ER.

In this post hoc analysis of pooled Phase 3 NB‐ER studies, medication adherence was examined at week 56 in patients who completed the COR studies. Among patients who reported taking ≥ 90% of dispensed medications, demographic and baseline clinical characteristics were evaluated as potential predictors of achieving percentage BW reduction and targeted anthropometrics at 56 weeks.

## 2 Methods

### 2.1 Study Design, Patients, and Treatment

This post hoc analysis was performed on pooled data from patients without diabetes who had completed any of the three Phase 3 COR trials (COR‐I, NCT00532779; COR‐II, NCT00567255; COR‐BMOD, NCT00456521) [22, 23, 24]. Patients who had a baseline BW measurement and a postbaseline BW measurement and were on study drug at week 56 were considered study completers. Patients with type 2 diabetes from the COR‐DM trial (NCT00474630) were not included in this analysis [25].

The three Phase 3, multicenter, randomized, double‐blind, and placebo‐controlled COR studies were previously described in detail [22, 23, 24]. Patients were eligible to enroll if they were aged 18–65 years with a BMI of ≥ 30 kg/m^2^ to ≤ 45 kg/m^2^ or a BMI ≥ 27 kg/m^2^ to ≤ 45 kg/m^2^ and controlled hypertension and/or dyslipidemia. In COR‐BMOD, patients randomized to either NB‐ER or placebo also received intensive group behavior modification. Drug compliance in each study was measured using the pill count at each visit after baseline. Across all studies, patients provided written informed consent, and the study protocols were approved by an institutional review board at each study site. All studies were conducted in accordance with the guidelines and principles of Good Clinical Practices standards and the Declaration of Helsinki.

The present analysis of the 3 COR studies included patients who were randomized to receive 32 mg naltrexone and 360 mg bupropion per day or placebo. Patients who received 16 mg naltrexone and 360 mg bupropion per day (COR‐I) and those who were re‐randomized to 48 mg naltrexone and 360 mg bupropion per day (COR‐II) were excluded from this analysis (*n* = 703).

### 2.2 Outcome Measures

This post hoc analysis first evaluated the overall percentage BW reduction and impact of cumulative medication adherence in patients who received NB‐ER or placebo. Furthermore, in patients adherent to NB‐ER across follow‐up, how baseline demographics and clinical characteristics (variables listed in Table 1) predicted changes in weight‐related health status targets after 56 weeks of treatment was assessed. Patients were considered achievers if they successfully reached defined weight‐related anthropometric targets of categorical BW reduction from baseline (≥ 5%, ≥ 10%, ≥ 15%), a race‐adjusted target BMI below the threshold of having clinical obesity (≤ 29.9 kg/m^2^; ≤ 24.9 kg/m^2^ for those reporting Asian heritage) [26, 27] and a race‐ and sex‐adjusted waist circumference below the very high obesity‐related health risk‐associated threshold (race: Black and European American, ≤ 88 cm [females], ≤ 102 cm [males]; Asian, ≤ 80 cm [females], ≤ 90 cm [males]) at 56 weeks of treatment [28, 29]. Nonachievers were defined as those who did not achieve these anthropometric targets at week 56.

### 2.3 Post Hoc Analysis Statistics

#### 2.3.1 Medication Adherence

Medication adherence was calculated by the number of pills taken divided by the number of pills prescribed and expressed as a percentage. Adherence values could exceed 100% if patients self‐increased doses during the titration period. General linear modeling was first used to assess if cumulative adherence at week 56 was associated with percent overall weight loss at week 56 in both NB‐ER and placebo trial completers. Among patients treated with NB‐ER, Pearson's Chi‐square tests were then used to analyze statistical differences between those who achieved or did not achieve anthropometric and BW reduction cutoff targets in those who had cumulative adherence to medication (≥ 90%) at week 56. In this analysis, “nonadherent” was defined as < 90% cumulative adherence to study medications at week 56.

#### 2.3.2 Univariable Analysis

In NB‐ER–treated trial completers with ≥ 90% adherence at week 56, baseline demographics and clinical characteristics were analyzed to predict response to treatment at week 56, indicated by achieving or not achieving defined categorical or target anthropometric indicators. Significant differences in baseline variables according to week 56 achievement of anthropometric and BW reduction cutoff point targets were determined by univariable logistic regression analysis. Estimated regression log‐odds coefficients were determined using a maximum likelihood estimates (MLE) model, which evaluated the impact of each predictor (demographic or clinical characteristic) on the likelihood of treatment response (achievement of the indicated health target) at week 56. For the population who self‐reported non‐Asian ancestry, baseline BMI classification was broken down into overweight (BMI ≥ 25 to ≤ 29.9 kg/m^2^), obesity class I (BMI ≥ 30 to ≤ 34.9 kg/m^2^), obesity class II (BMI ≥ 35 to ≤ 39.9 kg/m^2^), and obesity class III (BMI ≥ 40 kg/m^2^) categories. For the self‐reported Asian and South Asian ancestry population, baseline BMI was broken down into overweight (BMI ≥ 23 to ≤ 24.9 kg/m^2^) and obesity class I (BMI ≥ 25 kg/m^2^).

Crude odds ratios (ORs; exponential log‐odds coefficient) with 95% confidence intervals (CIs) of each independent baseline variable within the regression analysis were then calculated to determine the odds of achieving successful treatment target outcomes at week 56. An OR of one indicates no association between baseline predictor and treatment outcome.

#### 2.3.3 Multivariable Analysis

Within this same population, logistic regression modeling was then further used to determine how baseline demographics and clinical characteristics identified as predictors in the univariable analyses further contributed to predicting the likelihood of achieving a specified weight‐related target within a multivariable model. Only significant variables from the univariable analysis were included in the multivariable logistic regression analysis.

Similar to the univariable analysis, estimated regression log‐odds coefficients were determined under an MLE model. Wald Chi‐square tests were additionally run on all predictors to determine the statistical significance of each log‐odds coefficient. To adjust for small sample sizes and differences in BMI cutoff points for defined obesity classes across races, Firth's method, which adjusts the likelihood function to reduce bias in the estimates, was applied. This resulted in a “penalized” MLE model. Type III analysis of effects tests were run to determine if each baseline predictor of treatment (demographic or clinical characteristic) individually contributed to the prediction of the response in a linear regression model that accounted for all other variables already present.

ORs (95% CIs) of each predictor within the regression model were then calculated to determine the odds of achieving successful treatment target outcomes at week 56 dependent on respective demographics or clinical characteristics at baseline.

## 3 Results

### 3.1 Patient Baseline Demographics and Clinical Characteristics

**FIGURE 1:**
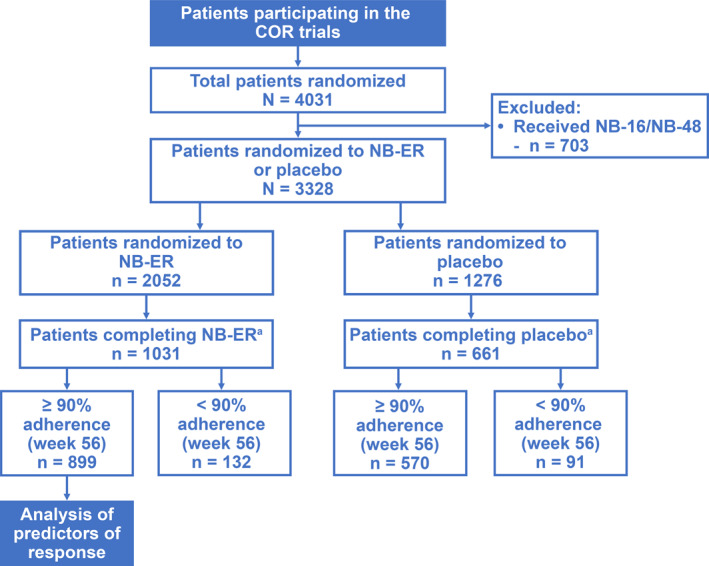
Post hoc analysis population. BW, body weight; NB‐ER, fixed‐dose, extended‐release combination of naltrexone and bupropion; NB‐16, fixed‐dose, extended‐release combination of 16 mg naltrexone and 360 mg bupropion; NB‐48, fixed‐dose, extended‐release combination of 48 mg naltrexone and 360 mg bupropion. ^a^Patients who had a baseline BW measurement and a postbaseline BW measurement while on study drug at week 56.

Of the 4031 total patients included in the 3 COR trials, 3328 were randomized to receive either placebo or NB‐ER and included in the present analysis (Figure 1). Of those patients, 1031/2052 (50.2%) completed the study on NB‐ER and 661/1276 (51.8%) completed the study on placebo. Baseline demographic and clinical characteristics for 1692 patients who were randomized to NB‐ER or placebo and completed a COR trial are summarized in Table 1. Overall, in the NB‐ER and placebo groups, respectively, the mean ages of patients were 46.0 and 46.2 years, and 83.6% and 83.5% were female. There were no significant differences between treatment groups by age, sex, or other demographics and baseline anthropometric measures. The majority of patients who received NB‐ER were categorized as having obesity class I or II at baseline (see breakdown of baseline demographic and clinical characteristics by obesity class in Supporting Information S1: Table S1).

**TABLE 1:** Baseline demographics and clinical characteristics in patients who completed a COR trial.

	NB‐ER (*n* = 1031)	Placebo (*n* = 661)	*p*‐value
Age^a^	46.0 (10.6)	46.2 (10.9)	0.731
Sex,^b^ *n* (%)			0.958
Female	862 (83.6)	552 (83.5)	
Male	169 (16.4)	109 (16.5)	
Race,^b^ *n* (%)			0.272
American Indian or Alaska Native	17 (1.6)	8 (1.2)	
Asian	7 (0.7)	6 (0.9)	
Black or African American	136 (13.2)	109 (16.5)	
Other	21 (2.0)	9 (1.4)	
White	850 (82.4)	529 (80.0)	
Ethnicity,^b^ *n* (%)			0.454
Hispanic or Latino	77 (7.5)	56 (8.5)	
Not Hispanic or Latino	954 (92.5)	605 (91.5)	
Years of education,^b^ *n* (%)			0.143
7–12	182 (17.7)	117 (17.7)	
13–16	618 (59.9)	426 (64.4)	
> 16	213 (20.7)	108 (16.3)	
N/A	18 (1.7)	10 (1.5)	
Income^b^			0.493
< $25,000	73 (7.1)	44 (6.7)	
$25,000 to ≤ $50,000	209 (20.3)	146 (22.1)	
> $50,000 to ≤ $75,000	206 (20.0)	136 (20.6)	
> $75,000	341 (33.1)	195 (29.5)	
N/A	0 (0.0)	1 (0.2)	
Subject refused	202 (19.6)	139 (21.0)	
Weight (kg)^a^	100.4 (16.2)	99.3 (15.0)	0.158
BMI (kg/m^2^)^a^	36.1 (4.3)	36.0 (4.2)	0.564
Obesity class,^b^ *n* (%)			0.481
BMI < 30	30 (2.9)	12 (1.8)	
BMI ≥ 30 and < 35	391 (37.9)	261 (39.5)	
BMI ≥ 35 and < 40	366 (35.5)	239 (36.2)	
BMI ≥ 40	244 (23.7)	149 (22.5)	
Waist circumference (cm)^a^	109.1 (11.236)	109.3 (11.870)	0.722
Pulse rate (bpm)^a^	71.27 (8.612)	71.00 (8.600)	0.530
FCI Carbo subscale score^a^	19.12 (5.541)	19.07 (5.759)	0.861
FCI Sweet subscale score^a^	20.64 (6.236)	20.70 (6.119)	0.846
IDS‐SR total score^a^	6.57 (5.458)	6.43 (5.113)	0.602
HOMA‐IR ratio^a^	3.35 (3.174)	3.33 (4.380)	0.900
IWQOL total (transformed) score^a^	69.78 (17.504)	72.39 (16.927)	0.003
Hypertension,^b^ *n* (%)			0.140
No	816 (79.1)	503 (76.1)	
Yes	215 (20.9)	158 (23.9)	
Dyslipidemia,^b^ *n* (%)			0.550
No	476 (46.2)	315 (47.7)	
Yes	555 (53.8)	346 (52.3)	
Impaired fasting glucose,^b^ *n* (%)			0.549
No	757 (73.4)	494 (74.7)	
Yes	274 (26.6)	167 (25.3)	
Systolic blood pressure,^b^ *n* (%)			0.100
< 100	35 (3.4)	20 (3.0)	
100–129	867 (84.1)	534 (80.8)	
≥ 130	129 (12.5)	107 (16.2)	
Complicated obesity,^b^ *n* (%)			0.720
No	451 (43.7)	295 (44.6)	
Yes	580 (56.3)	366 (55.4)	
Dyslipidemia medications at baseline,^b^ *n* (%)			0.232
No	376 (36.5)	221 (33.4)	
Yes	179 (17.4)	125 (18.9)	
Completed	1031 (100.0)	661 (100.0)	
Alcohol use,^b^ *n* (%)			0.244
No	541 (52.5)	366 (55.4)	
Yes	490 (47.5)	295 (44.6)	
Smoke use,^b^ *n* (%)			0.736
No	956 (92.7)	610 (92.3)	
Yes	75 (7.3)	51 (7.7)	
History of smoke use,^b^ *n* (%)			0.057
No	636 (61.7)	438 (66.3)	
Yes	395 (38.3)	223 (33.7)	
History of depression,^b^ *n* (%)			0.125
No	912 (88.5)	568 (85.9)	
Yes	119 (11.5)	93 (14.1)	
History of anxiety,^b^ *n* (%)			0.330
No	987 (95.7)	639 (96.7)	
Yes	44 (4.3)	22 (3.3)	
History of other psychiatric disorder,^b^ *n* (%)			0.708
No	1018 (98.7)	654 (98.9)	
Yes	13 (1.3)	7 (1.1)	
History of antidepressant use,^b^ *n* (%)			0.471
No	918 (89.0)	581 (87.9)	
Yes	113 (11.0)	80 (12.1)	
History of anxiolytic use,^b^ *n* (%)			0.199
No	1014 (98.4)	655 (99.1)	
Yes	17 (1.6)	6 (0.9)	
History of other psychotropic drug use,^c^ *n* (%)			0.808
No	1020 (98.9)	655 (99.1)	
Yes	11 (1.1)	6 (0.9)	

*Note:* Values are means (SD) or frequency counts (with percentages).

Abbreviations: BMI, body mass index; FCI, Food‐Craving Inventory; HOMA‐IR, Homeostatic Model Assessment for Insulin Resistance; IDS‐SR, Inventory of Depressive Symptomatology–Self Report; IWQOL, Impact of Weight on Quality of Life; N/A, not applicable; NB‐ER, fixed‐dose, extended‐release combination of naltrexone and bupropion; SD, standard deviation.

^a^ Analysis of variance (one‐way).

^b^ *X* ^*2*^‐test.

^c^ Fisher's Exact test (two‐way). Values shown are means (SD) or frequency counts (with percentages).

### 3.2 Medication Adherence to NB‐ER and Placebo and Achievement of Weight‐Related Anthropometric Targets

**FIGURE 2:**
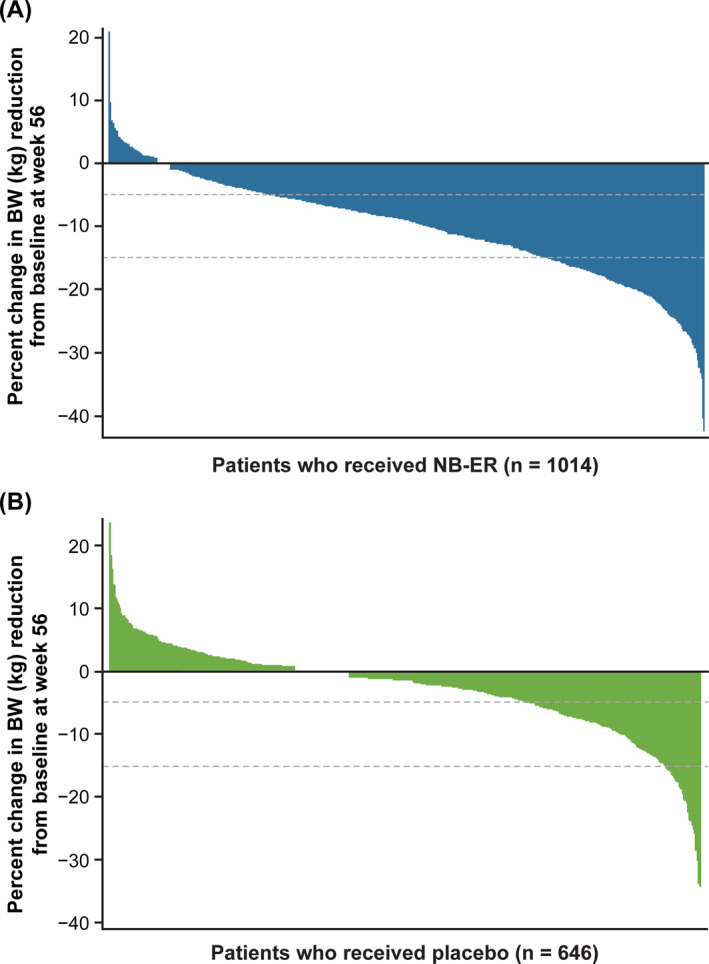
Waterfall plots of percent change in BW at week 56 in patients who had both baseline and week 56 BW measurements and received (A) NB‐ER^a^ or (B) placebo. Dotted lines represent −5% and −15% BW reduction. BW, body weight; NB‐ER, fixed‐dose, extended‐release combination of naltrexone and bupropion. ^a^Thirty‐two patients who completed the study did not have a week 56 BW measurement.

**FIGURE 3:**
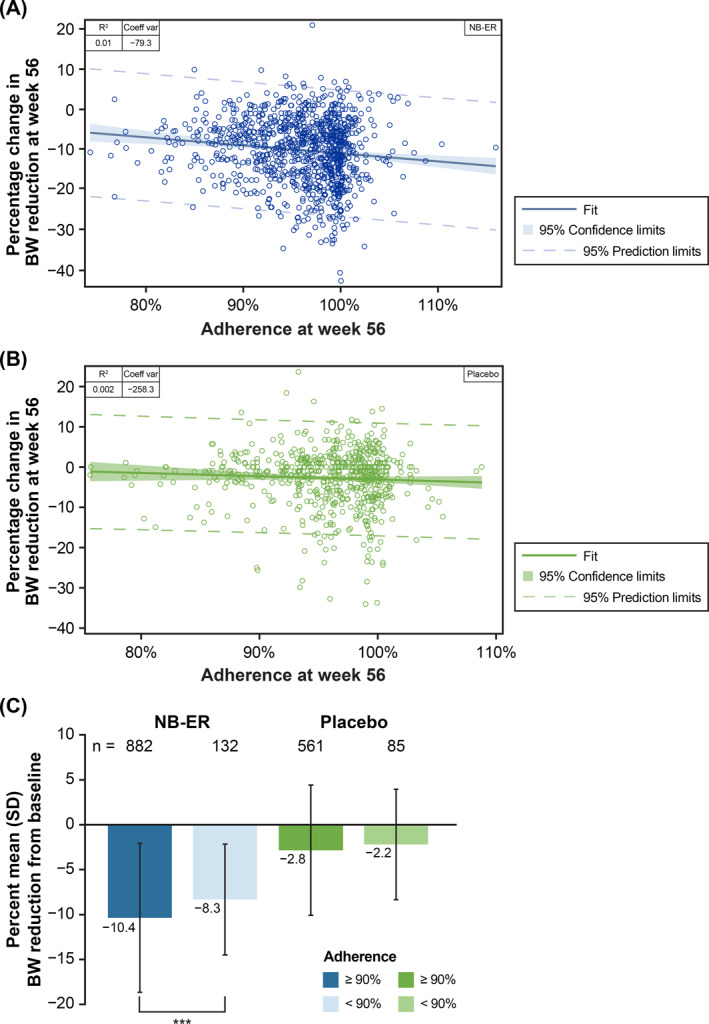
Fit model for the association between weight loss at week 56 and drug adherence at week 56^a^ in patients treated^b^ with (A) NB‐ER and (B) placebo. (C) Mean percent BW reduction in medication adherent versus nonadherent patients in NB‐ER and placebo groups. BW, body weight; Coeff Var, coefficient variable; NB‐ER, fixed‐dose combination of extended‐release naltrexone and bupropion; SD, standard deviation. *** denotes *p* < 0.001 as defined by a Student's *T*‐test. ^a^Adherence to treatment could exceed 100% if patients self‐increased doses during the titration period. ^b^Patients who had a BW measurement at week 56.

Among all patients who had both baseline and week 56 BW measurements, 91.8% (932/1014) versus 68.7% (444/646) of patients who received NB‐ER versus placebo demonstrated a reduction in BW from baseline, albeit with variability in the amount of BW reduction with both treatment assignments (Figure 2). At week 56, the majority of patients who completed the study and had a week 56 BW measurement were ≥ 90% adherent to treatment (NB‐ER, 87.0% [882/1014]; placebo, 86.8% [561/646]; 32 patients did not have a BW measurement recorded at week 56). Although patients receiving placebo were just as likely to be adherent to medication as patients who received NB‐ER, higher medication adherence at week 56 was associated with higher BW reduction for those on NB‐ER; this association was not observed for placebo (Figure 3A–B). Patients who were adherent to NB‐ER treatment demonstrated a significantly greater mean (SD) BW reduction of 10.4% (8.3%) compared with an 8.3% (6.2%) reduction among patients who received NB‐ER and were < 90% adherent (*p* = 0.0009; Figure 3C). Consistent with the absence of an observed significant association between adherence to placebo and BW reduction, patients ≥ 90% adherent versus < 90% adherent to placebo demonstrated similar, albeit non‐significant, percent reductions in BW at week 56 (2.8% [7.3%] vs 2.2% [6.1%]; *p* = 0.3868).

**FIGURE 4:**
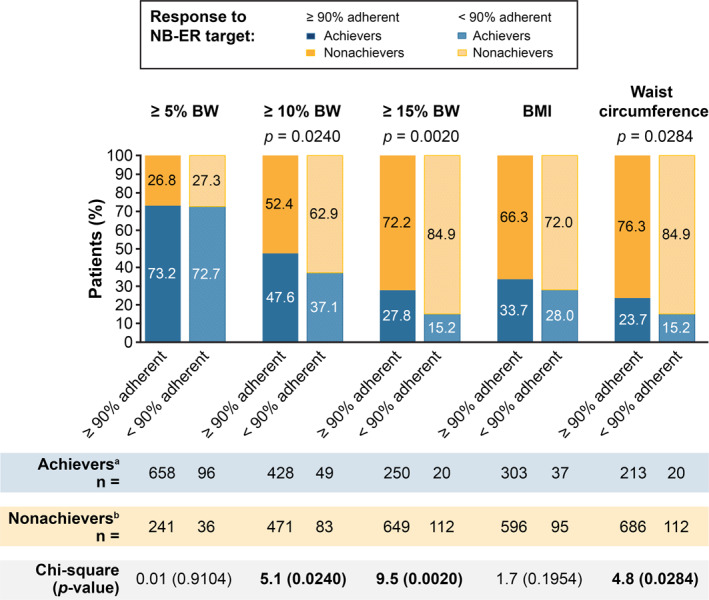
Percentage of achievers and nonachievers across week 56 weight‐related health targets by NB‐ER treatment adherence. BMI, kg/m^2^; BW, kg. Percentages of patients are within each bar section. Adherence was defined as ≥ 90% or < 90% adherence to NB‐ER at week 56. BMI, body mass index; BW, body weight; NB‐ER, fixed‐dose combination of extended‐release naltrexone and bupropion. ^a^Patients were considered achievers if they successfully reached defined weight‐related anthropometric targets of categorical BW reduction from baseline (≥ 5%, ≥ 10%, ≥ 15%), a race‐adjusted target BMI (race: Black and European American, ≤ 29.9 kg/m^2^; Asian, ≤ 24.9 kg/m^2^), or a race‐adjusted target waist circumference (race: Black and European American, ≤ 88 cm [females], ≤ 102 cm [males]; Asian, ≤ 80 cm [females], ≤ 90 cm [males]) at week 56 of treatment. ^b^Nonachievers were defined as those who did not achieve these anthropometric targets at week 56.

Among completers who received NB‐ER, ≥ 90% medication adherence also significantly impacted the achievement of categorical BW reduction and anthropometric targets (Figure 4). Adherence was significantly associated with achieving ≥ 10% BW (428/899 [47.6%], *χ* ^2^ = 5.092, *p* = 0.024) and ≥ 15% BW reduction (250/899 [27.8%], *χ* ^2^ = 9.5, *p* = 0.002) at week 56, though it was not associated with ≥ 5% BW reduction (658/899 [73.2%], *χ* ^2^ = 0.01, *p* = 0.9104). Furthermore, having ≥ 90% adherence at week 56 was significantly associated with achieving target waist circumference (303/899 [33.7%], *χ* ^2^ = 4.8, *p* = 0.028) but not with achieving target BMI (303/899 [33.7%], *χ* ^2^ = 1.7, *p* = 0.1954).

### 3.3 Univariable Analyses: Differences in Treatment Response by Baseline Demographic and Clinical Characteristics

Baseline demographics and clinical characteristics among those ≥ 90% adherent to NB‐ER associated with achieving ≥ 5% (Supporting Information S1: Figure S1A), ≥ 10% (Supporting Information S1: Figure S1B), ≥ 15% (Supporting Information S1: Figure S1C) BW reduction, target BMI (Supporting Information S1: Figure S1D), or target waist circumference (Supporting Information S1: Figure S1E) were identified by univariable analysis. Patients who were female versus male had higher odds (OR [95% CI]) of achieving BW reduction (≥ 5%, 1.94 [1.33–2.83]; ≥ 10%, 1.59 [1.10–2.30]; ≥ 15%, 1.78 [1.14–2.78]) and BMI targets (1.60 [1.07–2.41]). Conversely, male versus female patients were at better odds to achieve target waist circumference at week 56 (0.61 [0.41–0.91]). Patients who were Black versus White often had lower odds of achieving BW reduction cutoffs and anthropometric targets (≥ 5%, 0.47 [0.31–0.71]; ≥ 10%, 0.47 [0.31–0.72]; ≥ 15%, 0.35 [0.19–0.62]; BMI, 0.56 [0.35–0.90]; waist circumference, 0.55 [0.32–0.95]). Notably, female sex and White race represented the largest demographic categories of those enrolled in the COR trials. The number of patients in other race categories, including those with Asian ancestry, enrolled in the COR trials was small. Patients with higher education attainment (> 16 vs 7–12 years) also had higher odds of achieving > 15% BW reduction, BMI target, and waist circumference target at week 56 (≥ 15%, 1.63 [1.02–2.61]; BMI, 1.75 [1.13–2.72]; waist circumference, 1.70 [1.04–2.77]).

Higher baseline BW (kg), BMI (kg/m^2^), and waist circumference also predicted lower odds of achieving categorical ≥ 10% and ≥ 15% BW reduction and anthropometric targets at week 56. Furthermore, patients with obesity class II (BMI ≥ 35 to < 40) versus obesity class III (BMI ≥ 40) at baseline were at better odds to achieve ≥ 10% BW reduction, ≥ 15% BW reduction, and waist circumference targets (≥ 10%, 1.90 [1.32–2.72]; ≥ 15%, 1.75 [1.15–2.68]; waist circumference, 6.66 [2.60–17.06]). Patients who identified as non‐smokers at baseline had greater odds of achieving all weight‐related targets except for target waist circumference (≥ 5%, 1.77 [1.06–2.96]; ≥ 10%, 1.73 [1.04–2.90]; ≥ 15%, 2.68 [1.31–5.49]; BMI, 3.16 [1.59–6.27]). Among metabolic‐related clinical characteristics, baseline homeostatic model assessment for insulin resistance (HOMA‐IR) level predicted lower odds of achieving BMI or waist circumference targets but did not predict the odds of achieving BW reduction thresholds. Finally, patients who did not have impaired fasting blood glucose at baseline had greater odds of achieving ≥ 15% BW reduction and target waist circumference versus those who did (≥ 15%, 1.43 [1.01–2.02]; waist circumference, 1.46 [1.01–2.12]).

### 3.4 Multivariable Analysis: Predictors of Achieving Weight‐Related Targets

**FIGURE 5:**
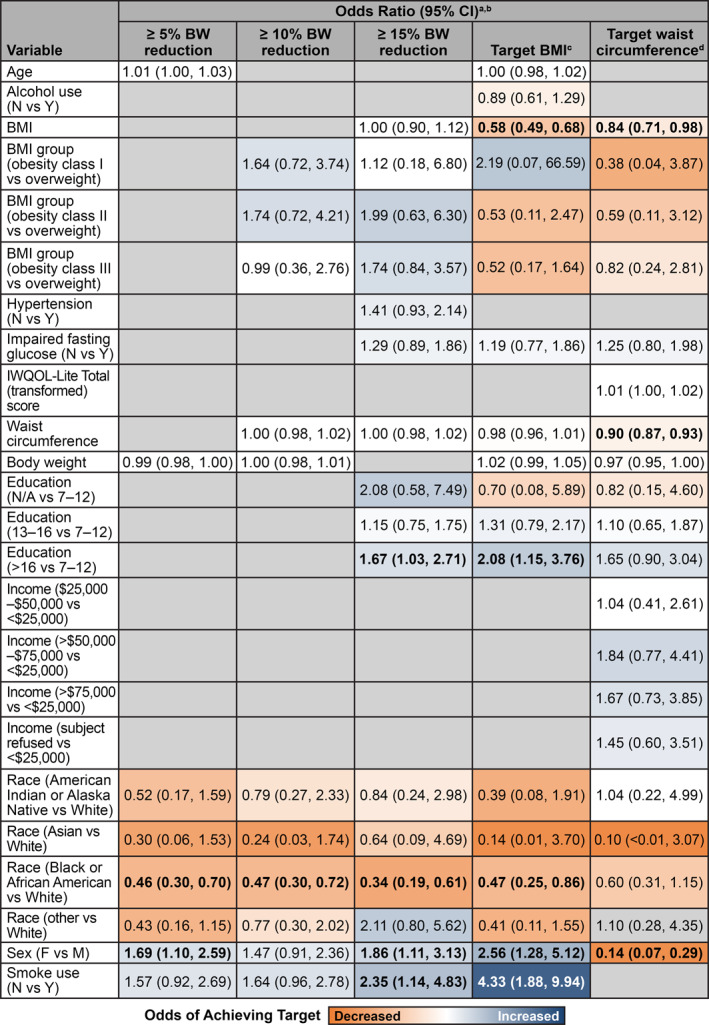
Multivariable analysis of baseline demographic and clinical characteristics predicting achievement of ≥ 5%, ≥ 10%, or ≥ 15% BW reduction, target BMI, and target waist circumference in trial‐completing patients who were ≥ 90% adherent to NB‐ER. BW, body weight; BMI, body mass index; CI, confidence interval; F, female; HOMA‐IR, homeostatic model assessment for insulin resistance; IWQOL‐Lite; Impact of Weight on Quality of Life‐Lite; M, male; N, no; N/A, not applicable; NB‐ER, fixed‐dose, extended‐release combination of naltrexone and bupropion; Y, yes. ^a^Bolded values indicate significant predictors (95% CI range does not contain 1.00). ^b^All significant variables in the univariable analysis were included in the multivariable analysis except HOMA‐IR at baseline due to ≥ 20 missing observations. ^c^Threshold is < 30 kg/m^2^, except in patients reporting Asian heritage, for whom the threshold is < 25 kg/m^2^. ^d^Thresholds are ≤ 88 cm in females and ≤ 102 cm in males for patients reporting as African or European American; for patients with Asian heritage, the waist circumference threshold is ≤ 80 cm in females and ≤ 90 cm in males.

Within a multivariable model, sex and race were the most consistent significant predictors of NB‐ER treatment response (Figure 5, see MLE and Type III analyses in Supporting Information S1: Tables S2–S7). Female sex was associated with increased odds (OR estimate [95% CI]) of achieving most weight‐related health targets (≥ 5%, 1.69 [1.10–2.59]; ≥ 15%, 1.86 [1.11–3.13]; BMI, 2.56 [1.28–5.12]). However, female patients had lower odds of achieving target waist circumference (0.14, 0.07–0.29). Additionally, Black versus White race was associated with lower odds of achieving most targets (≥ 5%; 0.46 [0.30–0.70]; ≥ 10%, 0.47 [0.30–0.72]; ≥ 15%, 0.34 [0.19–0.61]; BMI, 0.47 [0.25–0.86]). However, there was no association between patient race and meeting the waist circumference target. Higher educational attainment (> 16 years vs 7–12 years) significantly predicted ≥ 15% BW reduction and BMI target achievement (> 15%, 1.67 [1.03–2.71]; BMI, 2.08 [1.15–3.76]). Finally, although patient BW, obesity class, and waist circumference at baseline each independently predicted odds of successful treatment response within the univariable analysis, only baseline BMI maintained a significant relationship with lower odds of achieving BMI and waist circumference targets at week 56 (BMI, 0.58 [0.49–0.68]; waist circumference, 0.84 [0.71–0.98]).

## 4 Discussion

This pooled analysis of patients without diabetes treated with NB‐ER who completed 56 weeks of treatment showed that medication adherence at 56 weeks is an important predictor of BW reduction. Patients who were ≥ 90% adherent to NB‐ER at week 56 demonstrated significantly greater average BW reduction (10.4%) than those less adherent (8.3%). Further analyses among those with ≥ 90% adherence at week 56 showed that more patients treated with NB‐ER (91.7%) versus placebo (68.3%) lost weight from baseline. Notably, in the NB‐ER–treated medication‐adherent population, 47.6% and 27.8% achieved ≥ 10% and ≥ 15% BW reduction, respectively. Additionally, 33.7% and 23.7% of this population reached their target BMI and waist circumference, respectively. Even though medication adherence was associated with greater mean BW reduction, many with < 90% adherence were still able to achieve clinically significant BW reduction. Among the predictors of response to NB‐ER identified in this analysis, male sex and Black race predicted lower odds of achieving target anthropometric outcomes, whereas female sex, White race, higher education attainment, and negative smoking status at baseline were associated with greater odds of meeting target outcomes.

High medication adherence (≥ 90%) was associated with achieving greater BW reduction at week 56 but was not with BMI target attainment, highlighting the importance of evaluating multiple anthropometric measures when determining the success of OMs, as BMI does not directly reflect body composition or distribution of adiposity [8]. Higher adherence to treatment was beneficial to achieving higher categorical BW reduction (≥ 10% and ≥ 15% targets) and waist circumference reductions. Increased waist circumference is highly associated with visceral fat and mortality; therefore, long‐term treatment adherence may be most beneficial for patients with a high‐risk phenotype of obesity [30]. A secondary analysis of patients with obesity who received intensive behavioral therapy and were treated with liraglutide also found that medication adherence was associated with total weight loss at week 52 [31]. Similarly, patients from COR‐BMOD who received intensive group behavior modification with NB‐ER treatment were included in this post hoc analysis which demonstrated that adherence to medication predicted NB‐ER treatment response in a pooled clinical trial population. Patients who were ≥ 90% adherent at week 56 were more likely to achieve weight‐related targeted outcomes.

Low treatment adherence has been repeatedly associated with negative outcomes across a number of chronic conditions, including diabetes and cardiovascular disease [32]. Similarly, obesity management requires long‐term and comprehensive treatment that should include patient‐achievable goals and expectations [33, 34]. Patient adherence to treatment is multifaceted and can be defined by several metrics, including continued follow‐up over time, taking all doses of the prescribed medication, and following treatment instructions from the health care provider [32]. Here, adherence was assessed using cumulative adherence to prescribed doses through week 56. Within this analysis, cumulative adherence of ≥ 90% at week 56 was high (87%), showing that adherence was achievable for most patients who remained in the study. However, it should be noted that only participants who completed a trial were analyzed in the post hoc analysis, inherently selecting patients adherent to required clinic visits.

Since higher medication adherence was associated with achieving greater BW reduction, patients with ≥ 90% adherent to NB‐ER at week 56 were selected to investigate patient‐level predictors of response. Patient demographics such as sex and race were significant in determining the odds of achieving most weight‐related targets at week 56. Female patients had at least 69% greater odds of achieving categorical BW reduction (≥ 5% or ≥ 15% thresholds) and target BMI; males had greater odds of achieving target waist circumference by week 56 of treatment. Race (Black vs White race) significantly decreased the odds of achieving categorical BW reduction and target BMI. Patient race did not predict the odds of achieving the target waist circumference at week 56. In these analyses, only 16.4% of patients who received NB‐ER were male, 13.2% were Black, and 0.7% were Asian. Furthermore, it is noteworthy that a higher proportion of the patients who were Black had obesity class III at baseline compared with the proportion of patients who were White, which could have impacted associations even in multivariable analysis. While this analysis sought to examine anthropometric targets, one must acknowledge that those who start a BW reduction effort closer to a target are more likely to achieve that target. Future studies should assess these characteristics in a more heterogeneous population. Moreover, overall response to NB‐ER was variable across target outcomes (BW vs BMI vs waist circumference) and predicted by patient race and sex, further driving the need to evaluate OM treatment response based on a personalized “treat‐to‐target” approach.

Psychosocial factors such as socioeconomic status and stress likely play a role in weight‐related outcomes and may drive some of the associations highlighted from these analyses [35]. A recent systematic review identified possible predictors of weight loss across several studies of patients with obesity and overweight undergoing lifestyle interventions for the treatment of obesity [36]. Where race was associated with weight loss, Black patients tended to be less likely to achieve weight‐related outcomes [36]. In studies where education had significant associations with outcomes, higher educational attainment was a positive predictor of weight loss, which is also consistent with our findings. Conversely, male sex was a significant predictor of greater BW reduction in studies with only lifestyle interventions. In this analysis of NB‐ER as well as in reports of incretin mimetic–therapies used for weight management, female versus male patients were more likely to achieve weight‐loss targets, suggesting that females may have a greater benefit from certain OMs than males [13, 37, 38].

Several baseline clinical characteristics individually predicted odds of achieving weight‐related targets at week 56. However, only higher baseline BMI was associated with decreased odds of achieving target BMI and waist circumference but not with categorical BW reduction cutoffs within the multivariable model. Similarly, a recent post hoc analysis of the STEP 1 trial examining the efficacy and safety of semaglutide in patients with obesity or overweight found that patients had comparable rates of BW reduction regardless of baseline BMI [39].

Furthermore, patients who did not smoke at baseline had greater odds of achieving most weight‐related anthropometric targets compared with those who did. However, a separate post hoc analysis of the COR trials revealed that patients with obesity who smoke and were treated with NB‐ER versus placebo reported greater weight reduction at week 56, suggesting that NB‐ER still benefits people who smoke [40].

Although patients in this analysis were non‐diabetic, more patients with BMI ≥ 40 kg/m^2^ had HOMA‐IR levels ≥ 3.0 at baseline, revealing that metabolic factors may need to be considered when examining the associations found in this analysis. HOMA‐IR levels at baseline were not included in the multivariable analysis due to missing data; however, baseline HOMA‐IR was a negative predictor for achieving BMI and waist circumference targets at week 56 in a univariable analysis. These relationships should be explored further to determine the NB‐ER treatment response when controlling for insulin and glucose sensitivity in patients without diabetes.

A strength of the present study was the use of both univariable analyses to identify predictors of response at initial exploration and analyses that examine multiple variables simultaneously to uncover more complex relationships between variables and health‐related outcomes. However, a potential limitation resulting from the methodological approach was the use of regression modeling that did not adjust for multiple comparisons, which may increase the likelihood of spurious findings. Additionally, small and skewed sample sizes across categorical groups including sex and race may limit the generalizability of the results to a more diverse population and reduce the robustness of the observed associations. However, imbalanced group sizes can be better handled using the MLE process that was applied in this analysis. Sex and race were consistent predictors of treatment response across all multivariable outcomes, although it should be noted that the target BMI threshold used in this analysis does not account for sex. Furthermore, the evolving obesity treatment landscape recognizes the limitation of defining obesity with BMI alone [10, 15, 41]. This analysis highlights that examining this easy‐to‐monitor measure in combination with other anthropometric measurements can provide a holistic view of overall health.

Waist‐to‐height ratio and relative fat mass are also recommended anthropometric metrics to assess obesity that are relatively easy to determine in the clinic; however, they were not evaluated because their close association with waist circumference made it unlikely to add any meaningful information to this analysis [8]. More detailed assessments of body composition such as dual‐energy X‐ray absorptiometry (DEXA) scans or bioimpedance analysis would provide greater insight into the effect of NB‐ER on body composition [10]. While body composition was not examined in this post hoc analysis, body composition using DEXA scans was assessed in a sub‐study of patients from COR‐I. This study demonstrated that NB‐ER induced BW reduction and was associated with significant reductions in fat mass and increases in total percentage lean mass. Notably, as the original COR trials had limited patient diversity, future studies should prioritize more diverse study populations and incorporate additional metrics to assess excess adiposity that account for sex and race. While this 56‐week analysis is consistent in duration with similar studies with OMs, long‐term studies are needed to further understand the relationship between patient demographics and clinical characteristics and weight‐related outcomes. Overall, these factors should be considered when interpreting the conclusions from this post hoc analysis.

This analysis is a first step in identifying which patient populations are most likely to respond to treatment and informing the shared decision‐making process between patients and their healthcare team in determining the use of OMs. Moreover, evaluating clinical trial results of weight‐related outcomes (e.g., BMI, waist circumference) other than BW reduction alone may offer a more complete view of individuals' health status. As obesity can be polygenic in nature, future studies will also need to evaluate genetic variation alongside patient demographics and clinical characteristics as potential predictors of treatment response [42].

## 5 Conclusions

In this post hoc analysis, ≥ 90% medication adherence was associated with greater mean BW reduction in study completers who were treated with NB‐ER but not among those treated with placebo. In those treated with NB‐ER, nearly half of the adherent patients reduced their BW by at least 10%, and 34% and 24% of patients reached a more healthful target BMI and waist circumference, respectively. However, for those who had < 90% adherence and received NB‐ER, a clinically significant proportion still achieved significant BW reduction and met anthropometric targets. In patients who were ≥ 90% adherent to NB‐ER, univariable and multivariable analyses were performed to identify baseline characteristics that predict successful achievement of BW reduction and anthropometric targets at 1 year of treatment. Although baseline demographic characteristics such as sex and race were associated with treatment response, our overall ability to predict NB‐ER treatment based on patient demographics and clinical characteristics at baseline is still limited. These secondary results from the COR trials emphasize the role of adherence to medication in the achievement of clinically significant outcomes.

## Author Contributions

D.H.R. contributed to the analysis conceptualization. All authors were involved in reviewing the data and manuscript drafts and had final approval of the submitted and published versions

## Funding

This work was sponsored by Currax Pharmaceuticals LLC.

## Conflicts of Interest

**D.H.R.** declares having received consulting honoraria from Abbvie, Altimmune, Amgen, AstraZeneca, Biohaven, Boehringer Ingelheim, Calibrate, Carmot Therapeutics, Currax Pharmaceuticals LLC, CinRx, Eli Lilly, Epitomee, Fractyl Health Inc., I2o, ICON, Kailera, Novo Nordisk, Pfizer, PPD, Protagonist, Regor, Rhythm, Souffle, Source Bio, Structure Therapeutics, Tenvie, Viking Therapeutics Inc., WondrHealth, Weight Watchers, and Zealand Pharma. She has received stock options from Calibrate, Epitomee, and Ro Health. **A.F.** reports work on advisory boards for Currax Pharmaceuticals LLC, Eli Lilly, Ms.Medicine, Novo Nordisk, Rhythm Pharmaceuticals, SideKick Health, Seca, and VIVUS. **R.F.K.** reports serving on advisory boards or consulting for honoraria from Antag, AstraZeneca, Boehringer Ingelheim, Currax Pharmaceuticals LLC, Eli Lilly, Novo Nordisk, Structure, Viking, and Weight Watchers. He also reports receiving Honoria for lectures, presentations, and travel support for Boehringer Ingelheim, Currax Pharmaceuticals LLC, Medspace, Primary Care Education Consortium, and Vindico. **J.S.T.** reports an investigator‐initiated grant, on behalf of the University of Pennsylvania, from Novo Nordisk and reports receiving consulting fees from Currax Pharmaceuticals LLC. **C.D.S.** reports serving on advisory boards or consulting for honoraria from Boehringer Ingelheim, Currax Pharmaceuticals LLC, Eli Lilly, Novo Nordisk, Regeneron, and Weight Watchers, serving on a speakers bureau of Novo Nordisk and Eli Lilly, and has received research funds from Regeneron and Rhythm Pharmaceuticals. **C.M.A.** reports receiving grants paid to her institution from Patient‐Centered Outcomes Research Institute and GI Dynamics Inc., serving as the treasurer for the World Obesity Federation, and receiving consulting fees from Aardvark Therapeutics Inc., AbbVie, Altimmune, Arrowhead Pharmaceuticals, BioAge, Biolinq Incorporated, Caribou Biosciences, CinFina Pharma, Covidien LP, Cowen and Company, Currax Pharmaceuticals LLC, EPG Communication Holdings, Form Health, Fractyl Health Inc., Keros Therapeutics, Lilly USA, L‐Nutra, Mediflix, Metsera Inc., NeuroBo Pharmaceuticals, Neurocrine Biosciences, NodThera Limited, Nutrisystem, OptumRx, Pain Script Corporation, Palatin Technologies, Redesign Health, ReShape Lifesciences, Riverview School, Roman Health Ventures, Scholar Rock, Terns, Verily Life Sciences, Very, Vida Health, Viking Therapeutics Inc., Wave Life Sciences, WondrHealth, Xeno Biosciences, Zyversa Therapeutics.

## Supporting information


Supporting Information S1

## Data Availability

The data that support the findings of this study are available on request from the corresponding author. The data are not publicly available due to privacy or ethical restrictions.
