# Supplementary material for: Identifying Predictors of Weight‐Related Outcomes With Fixed‐Dose, Extended‐Release Naltrexone and Bupropion Among Treatment‐Adherent Patients in Phase 3 COR Trials: A Treatment Target Analysis
Source: Obes Sci Pract. 2026 Jun 16;12(3):e70159. doi: 10.1002/osp4.70159 (PMC13270069; doi:10.1002/osp4.70159)
Supplement: Supplementary file 1 — Supporting Information S1 [file OSP4-12-e70159-s001.docx]

# **Identifying Predictors of Weight-Related Outcomes With Fixed-Dose, Extended-Release Naltrexone and Bupropion Among Treatment-Adherent Patients in Phase 3 COR Trials: A Treatment Target Analysis**

**Authors:** Donna H. Ryan^1^, Angela Fitch^2^, Robert F. Kushner^3^, Jena S. Tronieri^4^, Christopher D. Still^5^, Caroline M. Apovian^6^

**Affiliations:** ^1^Pennington Biomedical Research Center, Baton Rouge, LA, USA; ^2^knownwell, Needham, MA, USA; **^3^**Northwestern University Feinberg School of Medicine, Chicago, IL, USA; ^4^University of Pennsylvania Perelman School of Medicine, Philadelphia, PA, USA; ^5^Geisinger Commonwealth School of Medicine, Danville, PA, USA; ^6^Brigham and Women’s Hospital Center for Weight Management and Wellness, Boston, MA, USA

**Supplemental tables and figures**

**Table S1.** Baseline demographics and clinical characteristics by overweight/obesity class in trial completers who received NB-ER

|  | **Overweight**  **(BMI <30 kg/m^2^)** | **Obesity class I**  **(BMI ≥30 and**  **<35 kg/m^2^)** | **Obesity class II**  **(BMI ≥35 and**  **<40 kg/m^2^)** | **Obesity class III**  **(BMI ≥40 kg/m^2^)** |
| --- | --- | --- | --- | --- |
|  | **NB-ER**  **(n = 30)** | **NB-ER**  **(n = 396)** | **NB-ER**  **(n = 364)** | **NB-ER**  **(n = 241)** |
| **Age, n (%)** |  |  |  |  |
| 18–24 | 0 | 8 (38.1) | 7 (33.3) | 6 (28.6) |
| 25–30 | 1 (1.6) | 22 (36.1) | 20 (32.8) | 18 (29.5) |
| 31–64 | 25 (3.1) | 318 (39.3) | 289 (35.7) | 178 (22.0) |
| 65+ | 1 (14.3) | 3 (42.9) | 3 (42.9) | 0 |
| **Sex, n (%)** |  |  |  |  |
| Female | 21 (2.8) | 296 (39.1) | 267 (35.3) | 173 (22.9) |
| Male | 6 (4.2) | 55 (38.7) | 52 (36.6) | 29 (20.4) |
| **Race, n (%)** |  |  |  |  |
| American Indian or Alaska Native | 1 (7.1) | 5 (35.7) | 4 (28.6) | 4 (28.6) |
| Asian | 0 | 6 (100.0) | 0 | 0 |
| Black or African American | 2 (1.8) | 40 (36.7) | 35 (32.1) | 32 (29.4) |
| Other | 0 | 10 (55.6) | 5 (27.8) | 3 (16.7) |
| White | 24 (3.2) | 290 (38.6) | 275 (36.6) | 163 (21.7) |
| **Ethnicity, n (%)** |  |  |  |  |
| Hispanic or Latino | 0 | 31 (45.6) | 23 (33.8) | 14 (20.6) |
| Not Hispanic or Latino | 27 (3.2) | 320 (38.5) | 296 (35.6) | 188 (22.6) |
| **Years of education, n (%)** |  |  |  |  |
| 7–12 | 5 (3.1) | 67 (41.6) | 56 (34.8) | 33 (20.5) |
| 13–16 | 14 (2.7) | 197 (37.4) | 187 (35.5) | 129 (24.5) |
| ≥16 | 6 (3.0) | 83 (42.1) | 71 (36.0) | 37 (18.8) |
| N/A | 2 (14.3) | 4 (28.6) | 5 (35.7) | 3 (21.4) |
| **Income, n (%)** |  |  |  |  |
| <$25,000 | 0 | 25 (39.1) | 23 (35.9) | 16 (25.0) |
| $25,000 to ≤$50,000 | 6 (3.4) | 54 (31.0) | 54 (31.0) | 60 (34.5) |
| >$50,000 to ≤$75,000 | 5 (2.7) | 73 (39.7) | 61 (33.2) | 45 (24.5) |
| >$75,000 | 10 (3.3) | 131 (43.1) | 111 (36.5) | 52 (17.1) |
| Subject refused | 6 (3.5) | 68 (39.3) | 70 (40.5) | 29 (16.8) |
| **Waist circumference group**, **n (%)** |  |  |  |  |
| Healthy | 11 (30.6) | 24 (66.7) | 1 (2.8) | 0 |
| Increased risk | 16 (1.9) | 325 (37.9) | 316 (36.9) | 200 (23.3) |
| **Weight group (kg), n (%)** |  |  |  |  |
| 0–99 | 27 (5.8) | 295 (63.0) | 137 (29.3) | 9 (1.9) |
| >99 | 0 | 56 (13.0) | 182 (42.2) | 193 (44.8) |
| **Complicated obesity, n (%)** |  |  |  |  |
| No | 2 (0.5) | 160 (40.9) | 144 (36.8) | 85 (21.7) |
| Yes | 25 (4.9) | 191 (37.6) | 175 (34.4) | 117 (23.0) |
| **Dyslipidemia, n (%)** |  |  |  |  |
| No | 4 (1.0) | 160 (39.3) | 150 (36.9) | 93 (22.9) |
| Yes | 23 (4.7) | 191 (38.8) | 169 (34.3) | 109 (22.2) |
| **Metabolic syndrome, n (%)** |  |  |  |  |
| No | 22 (3.4) | 266 (41.6) | 221 (34.5) | 131 (20.5) |
| Yes | 5 (1.9) | 85 (32.8) | 98 (37.8) | 71 (27.4) |
| **HOMA-IR, n (%)** |  |  |  |  |
| <2.0 | 12 (4.0) | 154 (51.3) | 91 (30.3) | 43 (14.3) |
| 2.0–2.9 | 8 (4.1) | 82 (42.3) | 74 (38.1) | 30 (15.5) |
| ≥3.0 | 5 (1.4) | 97 (27.3) | 135 (38.0) | 118 (33.2) |
| **Impaired fasting glucose subgroup, n (%)** |  |  |  |  |
| No | 19 (2.9) | 273 (41.0) | 225 (33.8) | 149 (22.4) |
| Yes | 8 (3.4) | 78 (33.5) | 94 (40.3) | 53 (22.7) |
| **Systolic blood pressure subgroup, n (%)** |  |  |  |  |
| ≤100 | 0 | 18 (66.7) | 3 (11.1) | 6 (22.2) |
| 100–129 | 22 (2.9) | 294 (38.6) | 268 (35.2) | 178 (23.4) |
| ≥130 | 5 (4.5) | 39 (35.5) | 48 (43.6) | 18 (16.4) |
| **Yypertension subgroup, n (%)** |  |  |  |  |
| No | 14 (2.0) | 302 (42.4) | 251 (35.2) | 146 (20.5) |
| Yes | 13 (7.0) | 49 (26.3) | 68 (36.6) | 56 (30.1) |
| **Alcohol use, n (%)** |  |  |  |  |
| No | 11 (2.3) | 167 (35.5) | 174 (37.0) | 118 (25.1) |
| Yes | 16 (3.7) | 184 (42.9) | 145 (33.8) | 84 (19.6) |
| **History of smoke use, n (%)** |  |  |  |  |
| No | 20 (3.6) | 220 (39.7) | 189 (34.1) | 125 (22.6) |
| Yes | 7 (2.0) | 131 (38.0) | 130 (37.7) | 77 (22.3) |
| **Smoke use, n (%)** |  |  |  |  |
| No | 26 (3.1) | 322 (38.7) | 300 (36.1) | 183 (22.0) |
| Yes | 1 (1.5) | 29 (42.6) | 19 (27.9) | 19 (27.9) |
| **History of depression, n (%)** |  |  |  |  |
| No | 26 (3.3) | 307 (38.7) | 281 (35.4) | 179 (22.6) |
| Yes | 1 (0.9) | 44 (41.5) | 38 (35.8) | 23 (21.7) |
| **History of antidepressant use, n (%)** |  |  |  |  |
| No | 26 (3.3) | 309 (38.6) | 282 (35.3) | 183 (22.9) |
| Yes | 1 (1.0) | 42 (42.4) | 37 (37.4) | 19 (19.2) |
| **History of anxiety, n (%)** |  |  |  |  |
| No | 26 (3.0) | 330 (38.3) | 309 (35.9) | 196 (22.8) |
| Yes | 1 (2.6) | 21 (55.3) | 10 (26.3) | 6 (15.8) |
| **History of anxiolytic use, n (%)** |  |  |  |  |
| No | 27 (3.0) | 343 (38.7) | 314 (35.4) | 202 (22.8) |
| Yes | 0 | 8 (61.5) | 5 (38.5) | 0 |
| **History of other psychiatric disorder, n (%)** |  |  |  |  |
| No | 27 (3.0) | 344 (38.7) | 318 (35.8) | 199 (22.4) |
| Yes | 0 | 7 (63.6) | 1 (9.1) | 3 (27.3) |
| **History of other psychotropic drug use, n (%)** |  |  |  |  |
| No | 27 (3.0) | 349 (39.2) | 314 (35.3) | 200 (22.5) |
| Yes | 0 | 2 (22.2) | 5 (55.6) | 2 (22.2) |

BMI, body mass index; HOMA-IR, Homeostatic Model Assessment for Insulin Resistance; N/A, not applicable; NB-ER, fixed-dose, extended-release combination of naltrexone and bupropion.

**Table S2.** Regression modeling of penalized maximum likelihood estimates of achieving ≥5% BW reduction at week 56 in patients ≥90% adherent to NB-ER treatment

|  |  | **≥5% BW** | | | |
| --- | --- | --- | --- | --- | --- |
| **Parameter** | **Category** | **Estimate** | **Standard error** | **Wald Chi−square** | ***P*-value** |
| **Intercept** |  | 0.2 | 0.7 | 0.09 | 0.7662 |
| **Weight** |  | −0.007 | 0.005 | 1.9 | 0.1687 |
| **Age** |  | 0.01 | 0.008 | 3.1 | 0.0779 |
| **Sex** | Female | 0.3 | 0.1 | 5.7 | 0.0170 |
| **Race** | Asian | −0.5 | 0.7 | 0.6 | 0.4567 |
| **Race** | American Indian or Alaska Native | 0.04 | 0.5 | 0.007 | 0.9325 |
| **Race** | Black or African American | −0.09 | 0.3 | 0.1 | 0.7426 |
| **Race** | Other | −0.1 | 0.4 | 0.1 | 0.7478 |
| **Smoke use** | N | 0.2 | 0.1 | 2.7 | 0.1009 |

BW, body weight; N, no; NB-ER, fixed-dose, extended-release combination of naltrexone and bupropion.

**Table S3.** Regression modeling of maximum likelihood estimates of achieving ≥10% BW reduction at week 56 in patients ≥90% adherent to NB-ER treatment

|  |  | **≥*10% BW*** | | | |
| --- | --- | --- | --- | --- | --- |
| **Parameter** | **Category** | ***Estimate*** | ***Standard error*** | ***Wald Chi−square*** | ***P−value*** |
| **Intercept** |  | -0.6 | 1.1 | 0.3 | 0.6068 |
| **Weight** |  | -0.004 | 0.009 | 0.2 | 0.6452 |
| **BMI subgroup** | Obesity class I | 0.2 | 0.1 | 2.5 | 0.1133 |
| **BMI subgroup** | Obesity class II | 0.3 | 0.1 | 4.1 | 0.0431 |
| **BMI subgroup** | Obesity class III | -0.3 | 0.2 | 1.4 | 0.2304 |
| **Sex** | Female | 0.2 | 0.1 | 2.5 | 0.1141 |
| **Race** | Asian | -0.9 | 0.8 | 1.2 | 0.2805 |
| **Race** | American Indian or Alaska Native | 0.3 | 0.5 | 0.4 | 0.5521 |
| **Race** | Black or African American | -0.2 | 0.3 | 0.6 | 0.4466 |
| **Race** | Other | 0.3 | 0.5 | 0.4 | 0.5354 |
| **Waist circumference** |  | -0.0004 | 0.01 | 0.002 | 0.9648 |
| **Smoke use** | N | 0.2 | 0.1 | 3.3 | 0.0683 |

BMI, body mass index; BW, body weight; N, no; NB-ER, fixed-dose, extended-release combination of naltrexone and bupropion.

**Table S4.** Regression modeling of maximum likelihood estimates of achieving ≥15% BW reduction at week 56 in patients ≥90% adherent to NB-ER treatment

|  |  | **≥15% BW** | | | |
| --- | --- | --- | --- | --- | --- |
| **Parameter** | **Category** | **Estimate** | **Standard error** | **Wald Chi−square** | ***P*-value** |
| **Intercept** |  | −2.2 | 2.0 | 1.1 | 0.2840 |
| **BMI subgroup** | Obesity class I | −0.2 | 0.5 | 0.2 | 0.6610 |
| **BMI subgroup** | Obesity class II | 0.4 | 0.2 | 2.5 | 0.1113 |
| **BMI subgroup** | Obesity class III | 0.2 | 0.2 | 1.2 | 0.2744 |
| **BMI** |  | 0.004 | 0.06 | 0.004 | 0.9507 |
| **Sex** | Female | 0.3 | 0.1 | 5.5 | 0.0191 |
| **Race** | Asian | −0.3 | 0.8 | 0.1 | 0.7561 |
| **Race** | American Indian or Alaska Native | 0.02 | 0.6 | 0.001 | 0.9742 |
| **Race** | Black or African American | −0.9 | 0.3 | 6.6 | 0.0103 |
| **Race** | Other | 0.9 | 0.5 | 4.1 | 0.0433 |
| **Waist** |  | 0.002 | 0.01 | 0.05 | 0.8313 |
| **Hypertension subgroup** | N | 0.2 | 0.1 | 2.7 | 0.1022 |
| **Impaired fasting glucose subgroup** | N | 0.1 | 0.09 | 1.9 | 0.1718 |
| **Smoke use** | N | 0.4 | 0.2 | 5.4 | 0.0201 |
| **Years of education** | 13–16 | −0.2 | 0.2 | 1.3 | 0.2611 |
| **Years of education** | >16 | 0.2 | 0.2 | 0.7 | 0.4067 |
| **Years of education** | N/A | 0.4 | 0.5 | 0.7 | 0.4143 |

BMI, body mass index; BW, body weight; N, no; N/A, not applicable; NB-ER, fixed-dose, extended-release combination of naltrexone and bupropion.

**Table S5.** Regression modeling of maximum likelihood estimates of achieving target BMI at week 56 in patients ≥90% adherent to NB-ER treatment

|  |  | **BMI** | | | |
| --- | --- | --- | --- | --- | --- |
| **Parameter** | **Category** | **Estimate** | **Standard error** | **Wald Chi−square** | ***P*-value** |
| **Intercept** |  | 16.5 | 2.8 | 35.3 | <0.0001 |
| **BMI subgroup** | Obesity class I | 0.9 | 1.1 | 0.6 | 0.4261 |
| **BMI subgroup** | Obesity class II | −0.5 | 0.4 | 1.6 | 0.2091 |
| **BMI subgroup** | Obesity class III | −0.5 | 0.4 | 1.7 | 0.1935 |
| **Weight** |  | 0.02 | 0.01 | 2.2 | 0.1 |
| **BMI** |  | −0.6 | 0.08 | 43.8 | <0.0001 |
| **Waist circumference** |  | −0.02 | 0.01 | 1.2 | 0.2726 |
| **Age** |  | 0.003 | 0.01 | 0.1 | 0.7551 |
| **Sex** | Female | 0.5 | 0.2 | 7.0 | 0.0080 |
| **Race** | Asian | −1.1 | 1.4 | 0.6 | 0.4372 |
| **Race** | American Indian or Alaska Native | −0.04 | 0.7 | 0.003 | 0.9602 |
| **Race** | Black or African American | 0.2 | 0.5 | 0.1 | 0.7380 |
| **Race** | Other | 0.02 | 0.7 | 0.001 | 0.9752 |
| **Years of education** | 13−16 | 0.1 | 0.3 | 0.1 | 0.7133 |
| **Years of education** | >16 | 0.6 | 0.3 | 3.4 | 0.0636 |
| **Years of education** | N/A | −0.5 | 0.8 | 0.4 | 0.5172 |
| **Impaired fasting glucose subgroup** | N | 0.09 | 0.1 | 0.6 | 0.4325 |
| **Alcohol use** | N | −0.06 | 0.1 | 0.4 | 0.5251 |
| **Smoke use** | N | 0.7 | 0.2 | 11.9 | 0.0006 |

BMI, body mass index; N, no; N/A, not applicable; NB-ER, fixed-dose, extended-release combination of naltrexone and bupropion.

**Table S6.** Regression modeling of maximum likelihood estimates of achieving target waist circumference at week 56 in patients ≥90% adherent to NB-ER treatment

|  |  | **Waist circumference** | | | |
| --- | --- | --- | --- | --- | --- |
| **Parameter** | **Category** | **Estimate** | **Standard error** | **Wald Chi−square** | ***P*-value** |
| **Intercept** |  | 17.8 | 2.9 | 37.7 | <0.0001 |
| **Weight** |  | −0.03 | 0.01 | 3.2 | 0.0756 |
| **BMI** |  | −0.2 | 0.08 | 4.8 | 0.0282 |
| **Sex** | Female | −1.0 | 0.2 | 27.8 | <0.0001 |
| **Race** | Asian | −1.7 | 1.4 | 1.5 | 0.2150 |
| **Race** | American Indian or Alaska Native | 0.6 | 0.7 | 0.6 | 0.4442 |
| **Race** | Black or African American | 0.02 | 0.5 | 0.001 | 0.9713 |
| **Race** | Other | 0.6 | 0.7 | 0.9 | 0.3550 |
| **Waist circumference** |  | −0.1 | 0.02 | 46.8 | <0.0001 |
| **IWQOL Total (transformed) score** |  | 0.009 | 0.006 | 2.6 | 0.1054 |
| **Impaired fasting glucose subgroup** | N | 0.1 | 0.1 | 1.0 | 0.3279 |
| **Income** | $25,000 to ≤$50,000 | −0.3 | 0.2 | 1.3 | 0.2467 |
| **Income** | >$50,000 to ≤$75,000 | 0.3 | 0.2 | 2.3 | 0.1276 |
| **Income** | >$75,000 | 0.2 | 0.2 | 1.4 | 0.2304 |
| **Income** | Subject refused | 0.06 | 0.2 | 0.1 | 0.7562 |
| **Years of education** | 13−16 | −0.002 | 0.2 | 0 | 0.9946 |
| **Years of education** | >16 | 0.4 | 0.3 | 2.2 | 0.1341 |
| **Years of education** | N/A | −0.3 | 0.6 | 0.2 | 0.6398 |
| **BMI subgroup** | Obesity class I | −0.5 | 0.6 | 0.8 | 0.3617 |
| **BMI subgroup** | Obesity class II | −0.1 | 0.3 | 0.1 | 0.7058 |
| **BMI subgroup** | Obesity class III | 0.2 | 0.3 | 0.8 | 0.3720 |

BMI, body mass index; IWQOL, Impact of Weight on Quality of Life; N, no; N/A, not applicable; NB-ER, fixed-dose, extended-release combination of naltrexone and bupropion.

**Table S7.** Regression modeling of Type III analysis of effects across all health status–related targets at week 56 in patients ≥90% adherent to NB-ER treatment

|  | **≥*5% BW*** | | **≥*10% BW*** | | **≥1*5% BW*** | | ***BMI*** | | ***Waist circumference*** | |
| --- | --- | --- | --- | --- | --- | --- | --- | --- | --- | --- |
| **Effect** | ***Wald Chi-square*** | ***P-value*** | ***Wald Chi-square*** | ***P-value*** | ***Wald Chi-square*** | ***P-value*** | ***Wald Chi-square*** | ***P-value*** | ***Wald Chi-square*** | ***P-value*** |
| **BMI** | N/A |  | 8.5 | 0.0363 | 0.003 | 0.9507 | 43.8 | <.0001 | 4.8 | 0.0282 |
| **Waist circumference** | N/A |  | 0.002 | 0.9648 | 0.05 | 0.8313 | 1.2 | 0.2726 | 46.8 | <.0001 |
| **Weight** | 1.9 | 0.1687 | 0.2 | 0.6452 | N/A |  | 2.2 | 0.1362 | 3.2 | 0.0756 |
| **Adherence to drug at week 28** | N/A |  | N/A |  | N/A |  | N/A |  | N/A |  |
| **Adherence to drug at week 56** | N/A |  | N/A |  | N/A |  | N/A |  | N/A |  |
| **Age** | 3.1 | 0.0779 | N/A |  | N/A |  | 0.1 | 0.7551 | N/A |  |
| **Pulse rate** | N/A |  | N/A |  | N/A |  | N/A |  | N/A |  |
| **IDS-SR total score** | N/A |  | N/A |  | N/A |  | N/A |  | N/A |  |
| **IWQOL total (transformed) score** | N/A |  | N/A |  | N/A |  | N/A |  | 2.6 | 0.1054 |
| **HOMA-IR score** | N/A |  | N/A |  | N/A |  | N/A |  | N/A |  |
| **Sex** | 5.7 | 0.0170 | 2.5 | 0.1141 | 5.5 | 0.0191 | 7.0 | 0.0080 | 27.8 | <.0001 |
| **Race** | 17.3 | 0.0017 | 13.8 | 0.0079 | 16.0 | 0.0030 | 9.5 | 0.0489 | 4.1 | 0.3935 |
| **Years of education** | N/A |  | N/A |  | 6.0 | 0.1140 | 6.9 | 0.0744 | 3.8 | 0.2848 |
| **Hypertension subgroup** | N/A |  | N/A |  | 2.7 | 0.1022 | N/A |  | N/A |  |
| **Dyslipidemia subgroup** | N/A |  | N/A |  | N/A |  | N/A |  | N/A |  |
| **Impaired fasting glucose subgroup** | N/A |  | N/A |  | 1.9 | 0.1718 | 0.6 | 0.4325 | 1.0 | 0.3279 |
| **Obesity class** | N/A |  | N/A |  | 4.8 | 0.1888 | 2.7 | 0.4344 | 1.0 | 0.7974 |
| **Income** | N/A |  | N/A |  | N/A |  | N/A |  | 4.5 | 0.3484 |
| **Alcohol use** | N/A |  | N/A |  | N/A |  | 0.4 | 0.5251 | N/A |  |
| **History of smoke use** | 2.7 | 0.1009 | 3.3 | 0.0683 | 5.4 | 0.0201 | 11.9 | 0.0006 | N/A |  |

BMI, body mass index; BW, body weight; IDS-SR, Inventory of Depressive Symptomatology–Self Report; IWQOL, Impact of Weight on Quality of Life; N/A, not applicable; NB-ER, fixed-dose, extended-release combination of naltrexone and bupropion.

**Figure S1.** Univariable analysis forest plots with odds ratios (95% CIs)^a^ of demographics and clinical characteristics at baseline predicting achievement of **(A)** ≥5%, **(B)** ≥10%, and **(C)** ≥15% BW reduction; **(D)** target BMI^b^; or **(E)** target waist circumference^c^ in patients who were ≥90% adherent to NB-ER^d^ at week 56

**(A)**

**
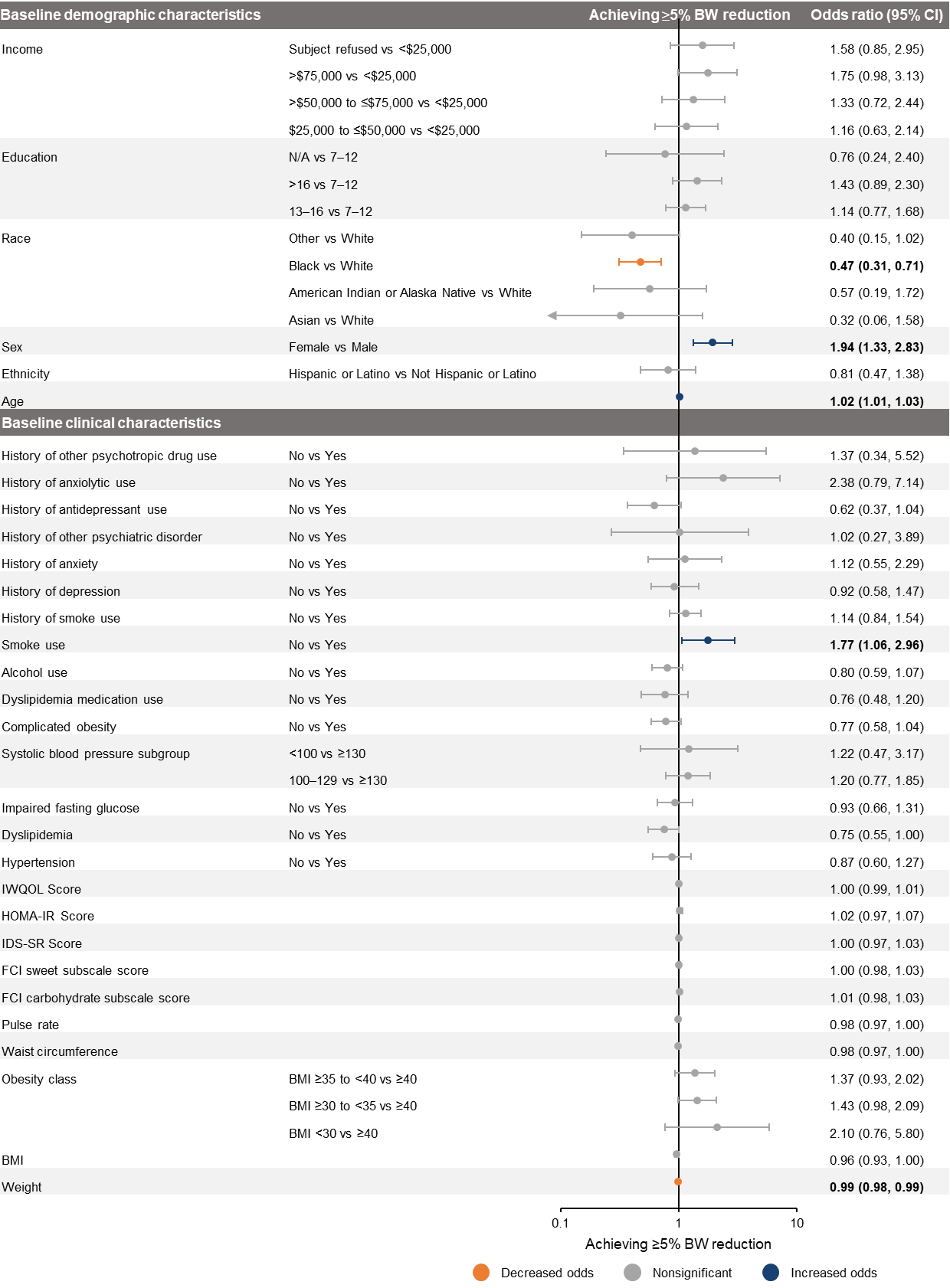
**

**(B)**

**
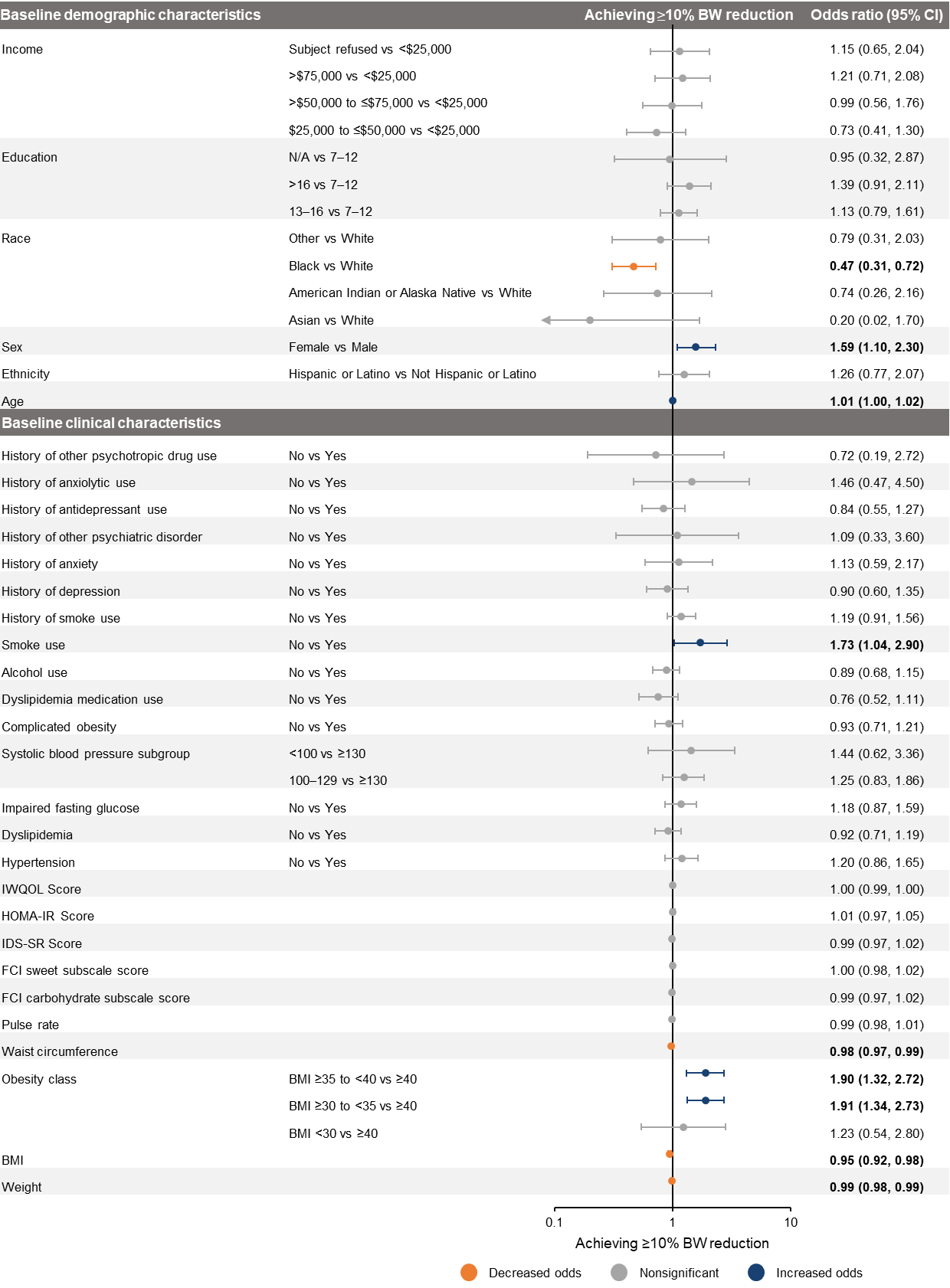
**

**(C)**

**
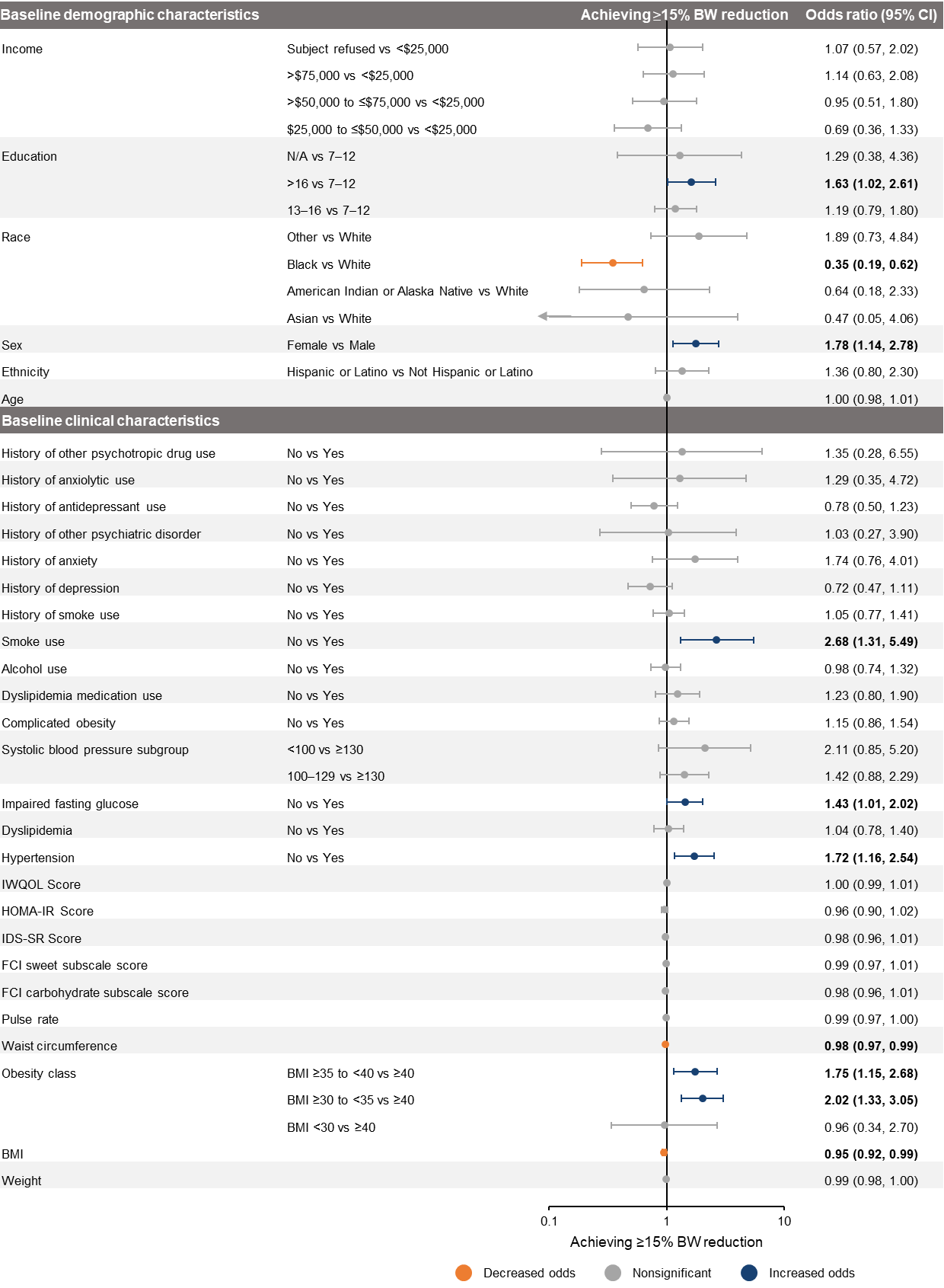
**

**(D)**

**
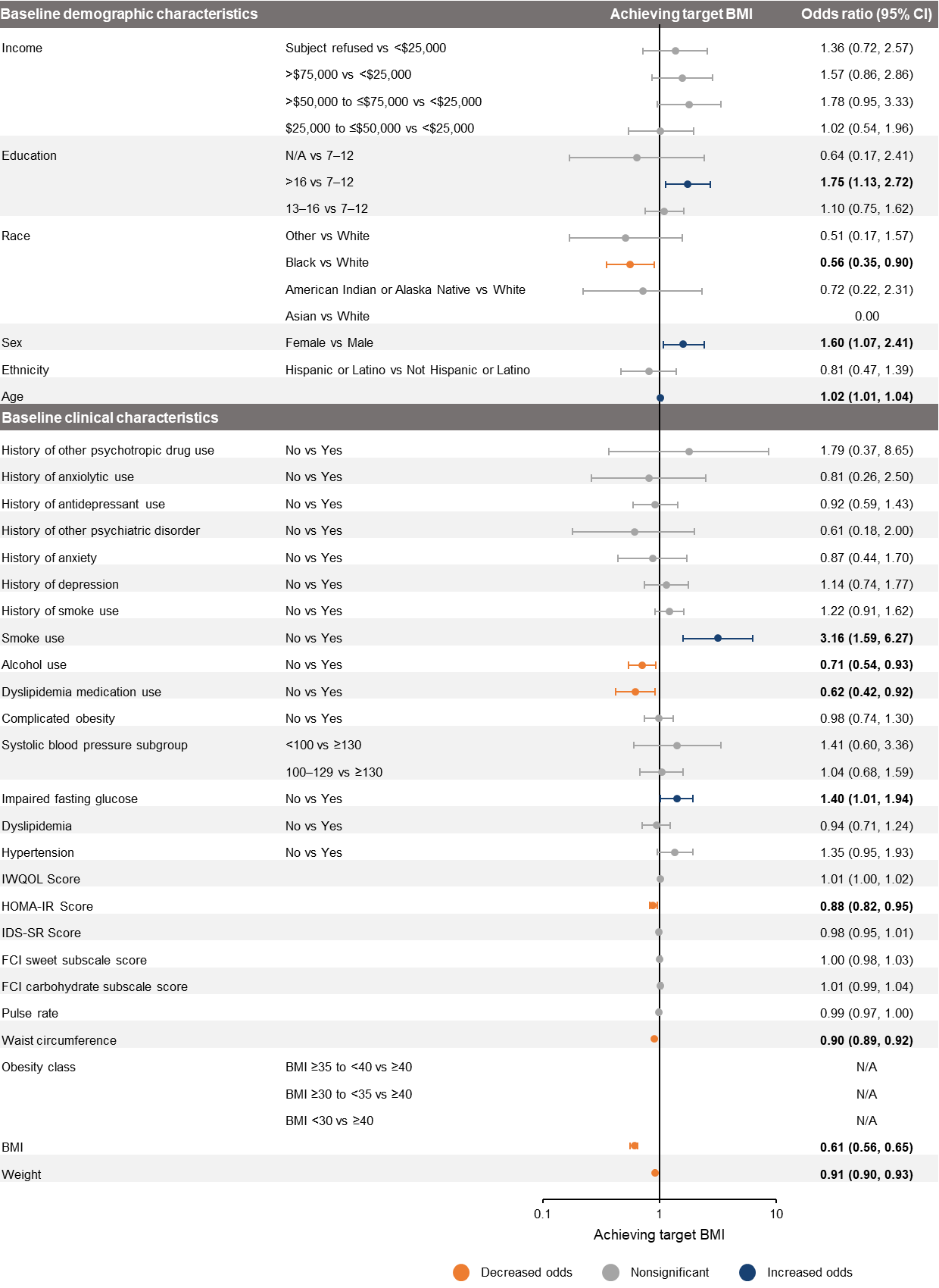
**

**(E)**

**
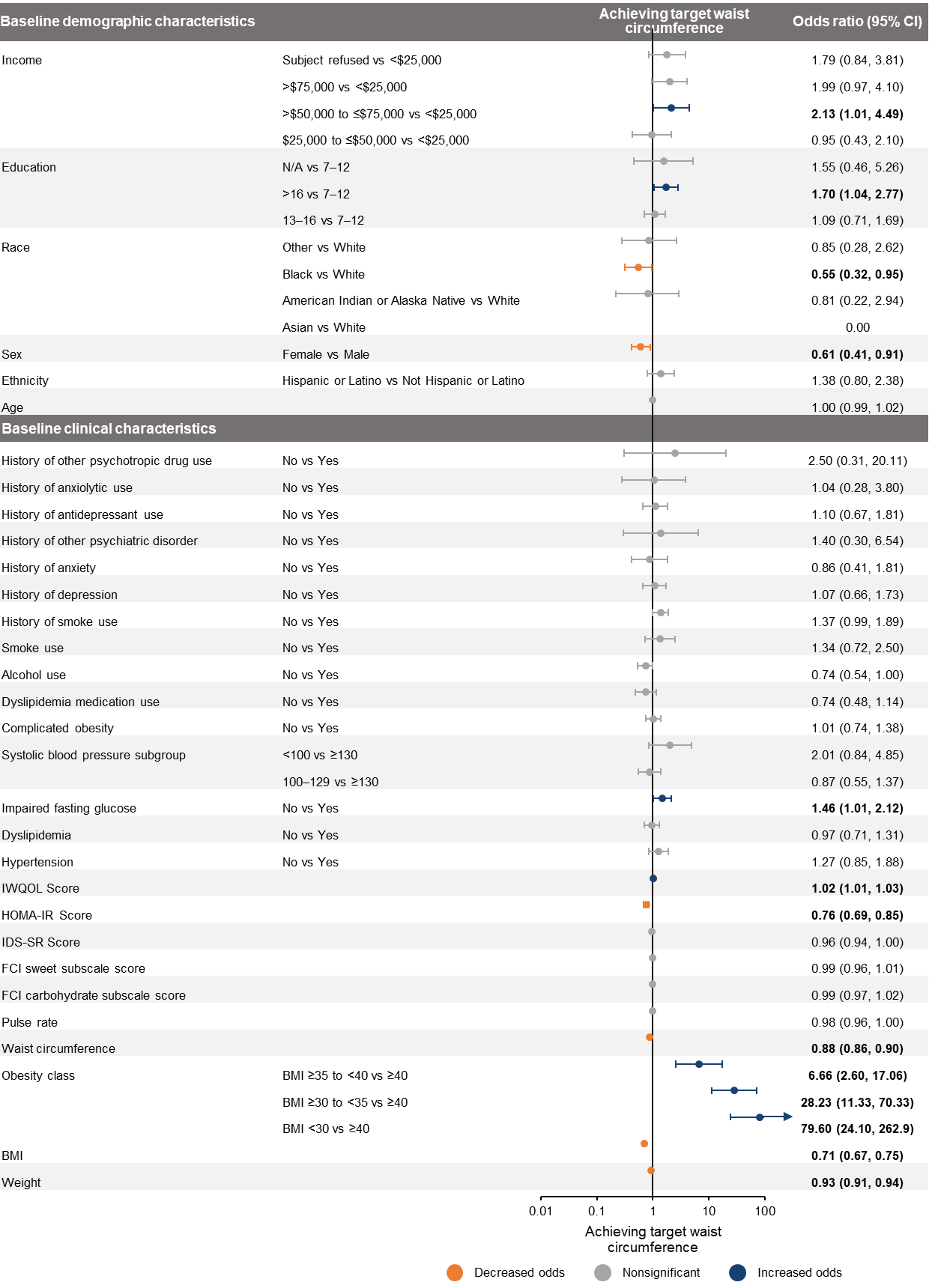
**

^a^Odds ratios were created from exponentiated estimated regression log-odds coefficients under an analysis of MLE model. To adjust for small sample sizes and differences in BMI cutoff points for defined obesity classes across races, Firth’s method to adjust the likelihood function to reduce bias in the estimates was applied. This resulted in a “penalized” MLE model.

^b^Threshold is <30 kg/m^2^, except in patients reporting Asian heritage, for whom the threshold is <25 kg/m^2^.

^c^Thresholds are ≤88 cm in females and ≤102 cm in males for patients reporting as African or European American; for patients with Asian heritage, the waist circumference threshold is ≤80 cm in females and ≤90 cm in males.

^d^n = 891; 8 patients were excluded due to missing data.

BMI, body mass index; BW, body weight; CI, confidence interval; F, female; FCI, Food Cravings Inventory; HOMA-IR, homeostatic measurement assessment of insulin resistance; IDS-SR, Inventory for Depressive Symtomatology–Self Report; IWQOL, Impact of Weight on Quality of Life; M, male; MLE, maximum likelihood estimated; N, no; N/A, not applicable; NB-ER, fixed-dose, extended-release combination of naltrexone and bupropion; Y, yes.
